# Successful Retrieval of an Embolized Vascular Closure Device (Angio-Seal^®^) After Peripheral Angioplasty

**DOI:** 10.1007/s00270-017-1565-9

**Published:** 2017-01-18

**Authors:** Philipp Jud, Rupert Portugaller, Dennis Bohlsen, Thomas Gary, Marianne Brodmann, Gerald Hackl, Franz Hafner

**Affiliations:** 1 Division of Angiology, Department of Internal Medicine, Medical University of Graz, Auenbruggerplatz 15, 8036 Graz, Austria; 2 Division of Vascular and Interventional Radiology, Department of Radiology, Medical University of Graz, Graz, Austria; 3 Division of Intensive Care, Department of Internal Medicine, Medical University of Graz, Graz, Austria

**Keywords:** Foreign body embolism, Vascular closure devices, Percutaneous transluminal angioplasty, Endovascular embolectomy, Peripheral artery disease

## Abstract

A 55-year-old male with peripheral arterial disease underwent angioplasty of the right lower limb arteries via antegrade femoral access. Angio-Seal^®^ closure device was used to treat the puncture site, whereby the intravascular sealing anchor accidentally embolized into the malleolar region of the right posterior tibial artery. Successful retrieval of the anchor was accomplished by a SpiderFX embolic protection device. This technique may be a useful approach to retrieve embolized foreign bodies via endovascular access.

## Introduction

Vascular closure devices aim to achieve hemostasis in patients who underwent an endovascular catheterization, enable an improved patient comfort compared to manual compression and reduce complications after endovascular access [1]. St. Jude Medical Angio-Seal^®^ represents one commonly used vascular closure device which is made from absorbable components including collagen sponge, polymer anchor and self-tightening suture. Due to an intra-arterial component of this vascular closure device, rare complications like infections, allergic reactions, anchor fracture and embolism may occur. We want to present one case of an embolized polymer anchor which could be successfully retrieved by using a SpiderFX^®^ embolic protection device.

## Case Report

A 55-year-old male patient presented to the outpatient clinic of vascular medicine due to an ulcer localized at the right metatarsophalangeal joint of the fifth toe. Magnetic resonance angiography of the pelvic and leg arteries revealed multiple stenoses of the right superficial femoral artery, the tibioperoneal trunk with a single-vessel runoff via the posterior tibial artery. Hence, we decided to perform a peripheral angioplasty of the right leg.

**Fig. 1:**
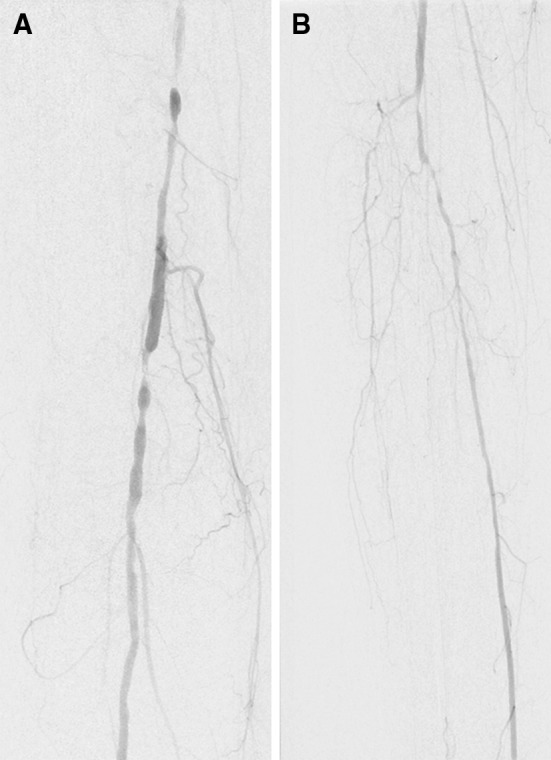
**A** Digital subtraction angiography of the right superficial femoral artery and popliteal artery with multiple stenoses before angioplasty. **B** Digital subtraction angiography of the crural arteries with several stenoses in the right anterior and posterior tibial artery and an occlusion of the fibular artery

**Fig. 2:**
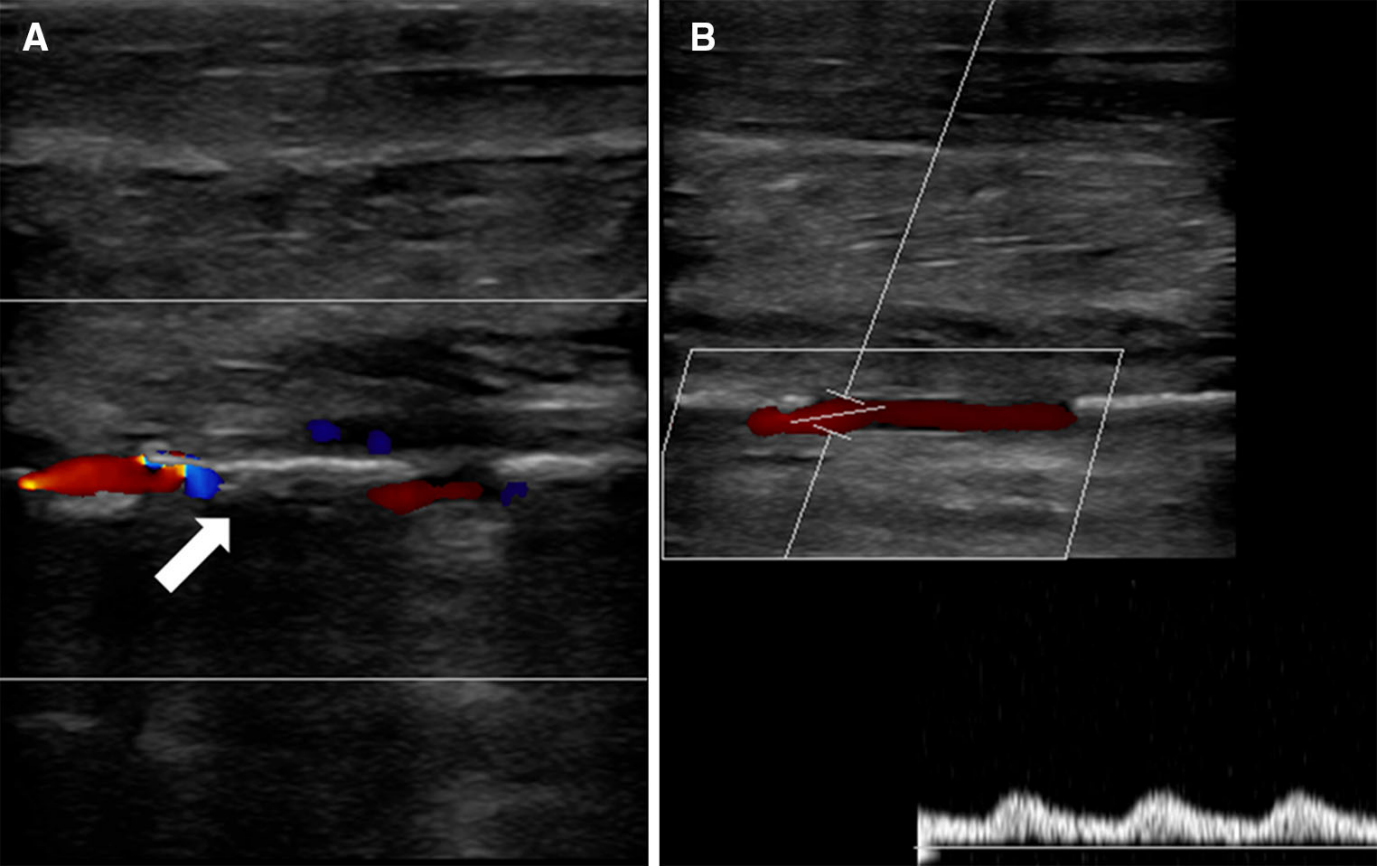
**A** Color-coded duplex ultrasonography of the proximal right posterior tibial artery. The *arrow* points toward the embolized Angio-Seal anchor. **B** Color-coded duplex ultrasonography displays the second segment of the right posterior tibial artery with a postobstructive blood flow

Via antegrade percutaneous access in the right groin, a six French introducer sheath was inserted with ultrasound guidance into the right common femoral artery. The appropriate access site was finally confirmed with fluoroscopic control. Angiography confirmed multisegmental high-grade stenoses of the right superficial femoral artery, popliteal artery and the tibioperoneal trunk with a one-vessel runoff via the posterior tibial artery (Fig. 1A, B). Femoro-popliteal lesions were treated using drug-coated balloons, whereas angioplasty with plain balloons was performed for infrapopliteal lesions. After removal of all devices, an Angio-Seal^®^ St. Jude Medical device was used to close the vascular puncture site according to the manufacturer’s instructions for use. After placing the arteriotomy locator/insertion sheath and inserting the carrier tube into the insertion sheath, we pulled on the device handle until resistance from the anchor catching on the distal tip of the insertion sheath was felt. The device handle was pulled back slowly until hemostasis was achieved and the suture was cut below the skin level. The patient had an extraordinary thin body constitution. Unfortunately, the cut was performed within the collagen sponge which led to embolization of the anchor. As hemostasis was no longer sufficient, manual compression for 15 min and a compression bandage were applied to the puncture site. Postinterventional color-coded duplex ultrasonography of the affected leg confirmed an obstruction of the proximal posterior tibial artery due to the embolized polymer anchor of the Angio-Seal^®^ closure device (Fig. 2A, B). The patient continued with the already prior to the intervention given antiplatelet therapy of 75 mg clopidogrel daily. Furthermore, we started therapeutic anticoagulation with low molecular weight heparin (enoxaparin one mg per kg body weight twice daily).

**Fig. 3:**
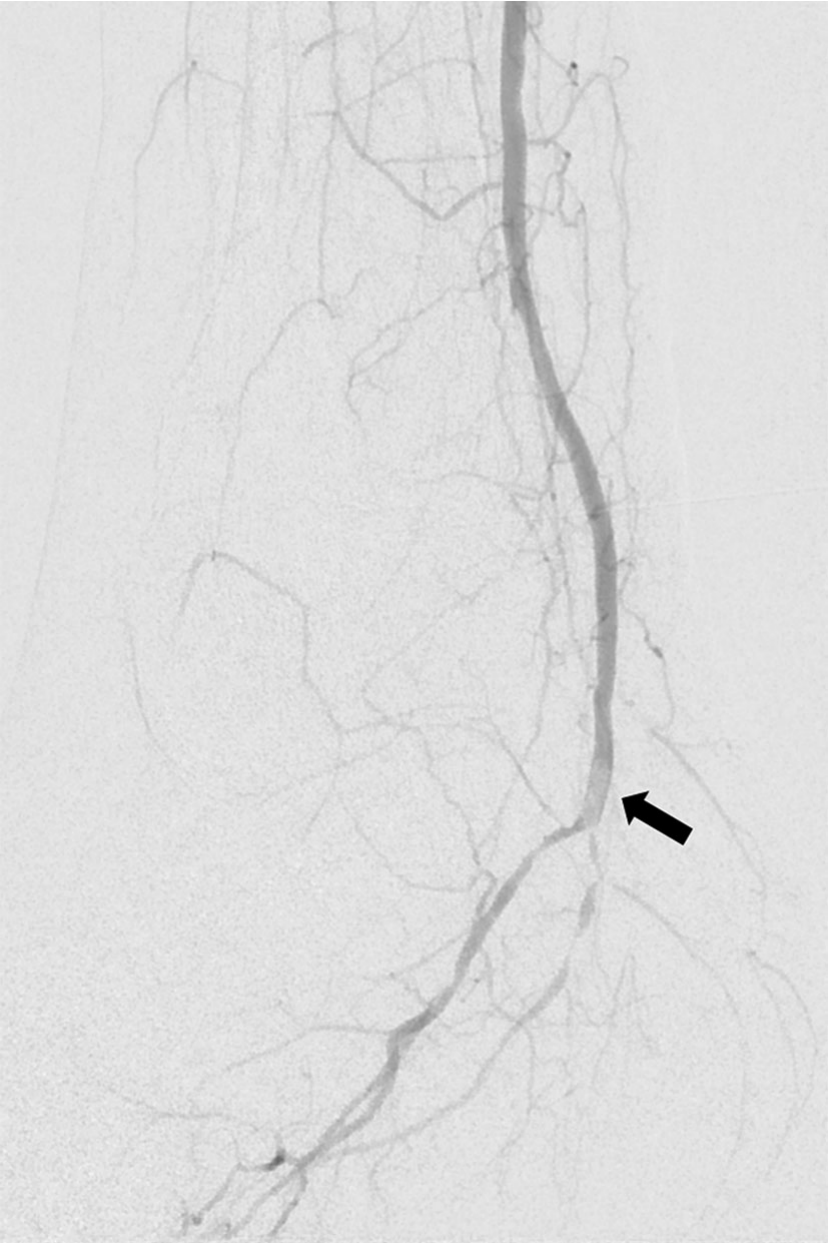
Digital subtraction angiography of the right posterior tibial artery at ankle’s level with a filling defect of approximately 8 × 3 mm

**Fig. 4:**
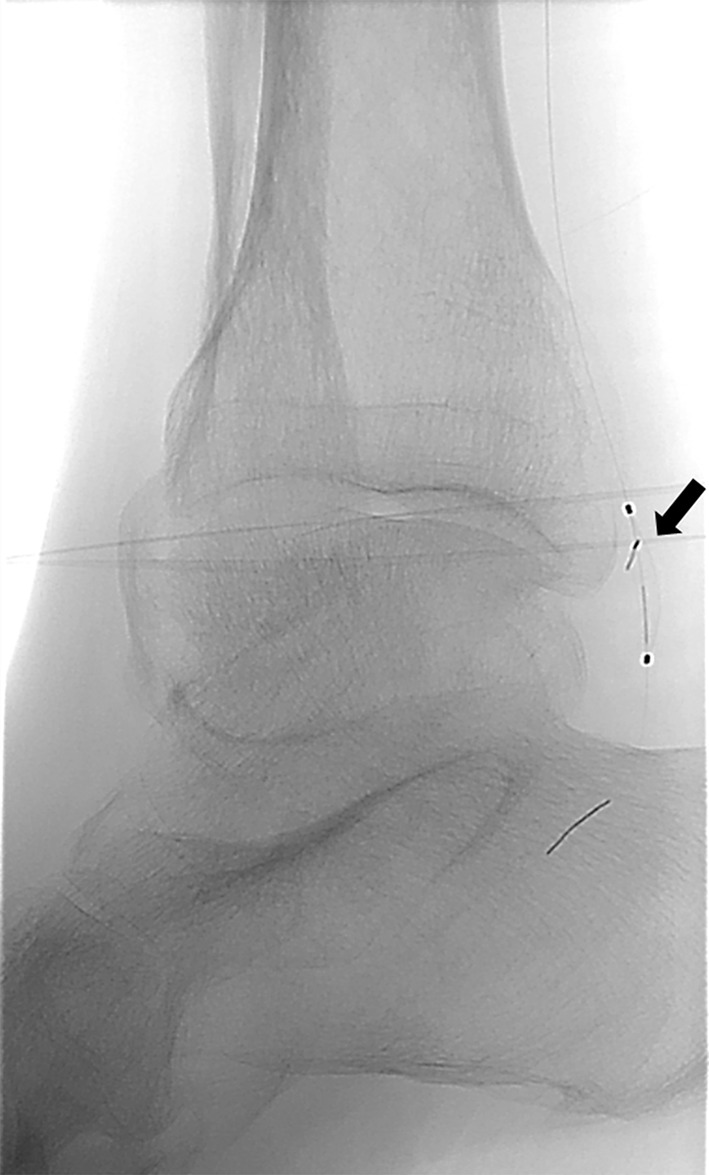
Release of the 4-mm SpiderFX filter system distal of the sealing anchor. The arrow points to the 4-mm SpiderFX filter system

No newly emerged symptoms occurred in the patient after that embolism. As the obstruction seemed hemodynamically relevant, we decided toward an endovascular embolectomy approach. After ultrasound-guided repeated percutaneous access in the right groin, we inserted a six French introducer sheath into the right common femoral artery. In contrast to the preceding vascular imaging by duplex ultrasonography and MR, the right proximal posterior tibial artery was patent, but a new filling defect was observed in the right distal posterior tibial artery at the level of the ankle (Fig. 3). That new filling defect corresponded to a further distal embolization of the Angio-Seal^®^ polymer anchor. In order to evaluate the anchor’s best retrieving method, we extracted the anchor of another sterile Angio-Seal^®^ device and tried to aspirate the anchor from a water-filled cup using different aspiration catheters of various size (four to seven French). The curved configuration of both anchor ends prevented a successful aspiration of the anchor independent to the size of the aspiration catheter. Therefore, we decided to retrieve the anchor using a filter system. After passing the Angio-Seal^®^ anchor with a 0.014-inch guide wire, a 4-mm SpiderFX^®^ embolic protection device was released distally to the embolized anchor (Fig. 4). The small size of the filter was selected because of the small reference vessel diameter of the posterior tibial artery. The filter system was pulled back, and the sealing anchor retrieval was successful. Vasospasm was observed in the postinterventional angiography control which was successfully treated with intra-arterial application of 10 mg nimodipine and subsequent intravenous prostanoid infusion over the next 48 h. Unfractionated heparin was administered under control of activated clotting time for 48 h after sheath removal. No further complications occurred. Postinterventional color-coded duplex ultrasonography confirmed a patent posterior tibial artery of the affected side, especially at the malleolar region.

## Discussion

Peripheral angioplasty is a minimal-invasive revascularization method in patients with peripheral artery occlusive disease. Peripheral embolism represents one of its complications. The incidence rate of clinically significant emboli in peripheral angioplasty ranges from 2.4 to 27.6% [2, 3]. However, embolism from foreign body of a vascular closure device is quite rare. Schroeder et al. reported of an embolism of a hemostasis valve rubber disk after coronary angioplasty, and a VasoSeal^®^ plug caused an acute occlusion of the right popliteal artery in the case report of Stiel et al. In both cases the embolized structures were retrieved surgically [4, 5].

**Fig. 5:**
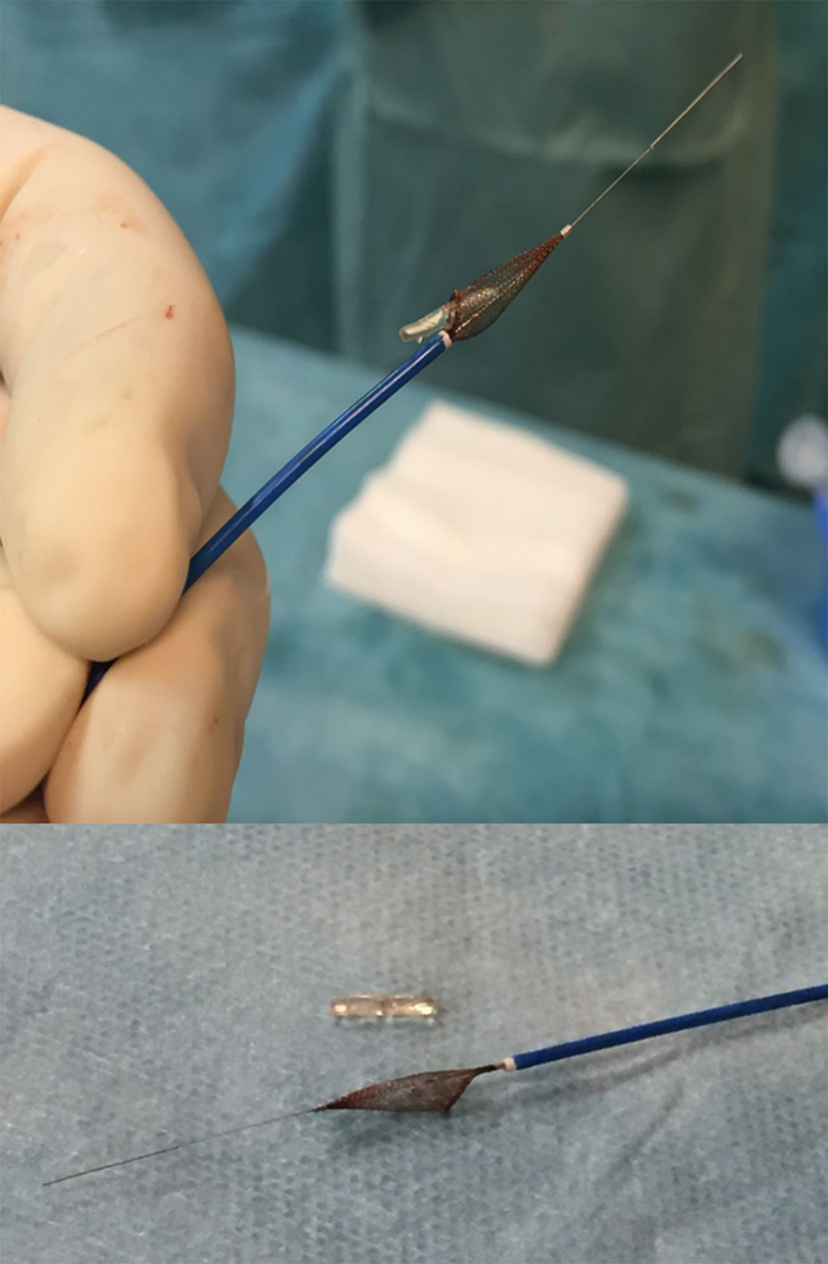
Embolized Angio-Seal device captured within the filter

Angio-Seal^®^ represents a commonly used vascular closure device in interventional radiology [6]. The use of such closure devices reduces the recovery time in the cath-lab and possibly increases turnover [7]. However, several adverse events may occur as previously reported and stated in the manufacturer’s instructions for use. An occlusion of the femoral artery may occur in 0.3–0.4% [8]. In one case, an Angio-Seal^®^ device even caused a foot ulcer by arterial obstruction at the puncture site, which was also removed surgically [9]. While these previously reported complications occurred at the puncture site, this case differs as it was located peripherally in the extremity. Peripheral embolization of the anchor may be related to anchor fracture, device malfunction or inappropriate detachment of the anchor. In our case, such an inappropriate detachment was related to a deeper cutting of the suture within the collagen sponge. We reported that this patient had a very thin body constitution; for this reason we were unable to properly tamp the collagen plug and knot, and therefore, the suture was cut within the collagen plug. As per the instructions for use, there are no contraindications as such, especially relating to antegrade punctures. Angio-Seal deployment is reported to be successful for both antegrade and retrograde femoral punctures albeit with a higher antegrade failure rate [10]. Antegrade access is not contraindicated. However, we believe that even a retrograde access would have had the same risk due to the thin body constitution. The patient was fortunately asymptomatic the entire time postembolism. However, duplex ultrasonography revealed a hemodynamically significant vascular obstruction due to the embolized anchor. Keeping in mind the limited vascular outflow in a patient with diabetes and several additional vascular risk factors and the presence of an arterial foot ulcer of the affected side, we aimed to improve arterial perfusion by retrieving the embolized anchor. Treatment options in such a case include endovascular retrieval or surgical intervention. Because of the previous arterial puncture of the right common femoral artery and the missing evidence of thrombotic complications, we decided against intra-arterial thrombolysis and toward an endovascular approach performing percutaneous embolectomy. Boersma et al. [11] reported successful retrieval of a dislodged femoral arterial closure device with an alligator forceps. Due to the small vessel diameter of the posterior tibial artery, we could not consider a similar retrieval technique and therefore decided to use a filter system. In case of a fail of the endovascular approach, surgical intervention would still have been an option. There are limited data on endovascular retrieval of an isolated Angio-Seal^®^ anchor without thrombus or collagen sponge. Abi Rafeh et al. [12] reported successful endovascular extraction of newer generation Angio-Seal^®^ collagen plug and anchor after acute embolization using a Spider^®^ RX 7-mm filter device (Covidien, Plymouth, MN, USA). We considered aspiration of the anchor and evaluated therefore aspiration catheters of various sizes. Unfortunately, the bent ending of the anchor prohibited successful foreign body aspiration. Finally, the anchor was successfully retrieved using a 4-mm SpiderFX^®^ embolic protection device. In contrast to the method published by Rafeh and colleagues, who placed the filter distally and inflated four French over the wire Fogarty balloon proximally and pushed the embolized anchor forward into the filter system, we tried to retract the opened filter, thereby catching the embolized anchor into the filter system. With that method, we successfully achieved endovascular foreign body embolectomy of the posterior tibial artery (Fig. 5). The advantages of this method are the minimal-invasive approach, the relatively small minimum size of the access sheath and good feasibility even in distal and smaller arteries. One disadvantage of this method could be a higher rate of vasospasm, which could be treated with a vasodilator, such as nimodipine as was successfully performed in our case.

## Conclusion

We want to remind physicians about the risk of peripheral embolism when using a vascular closure device with an intraluminal anchor like Angio-Seal^®^. Foreign body embolism of a vascular closure device is a very rare complication. We provide a feasible guidance for a minimal-invasive endovascular retrieval of such an embolized Angio-Seal^®^ anchor using a SpiderFX^®^ embolic protection device.
